# Risk Factors for Ocular Chlamydia after Three Mass Azithromycin Distributions

**DOI:** 10.1371/journal.pntd.0001441

**Published:** 2011-12-13

**Authors:** Berhan Ayele, Teshome Gebre, Jeanne Moncada, Jenafir I. House, Nicole E. Stoller, Zhaoxia Zhou, Travis C. Porco, Bruce D. Gaynor, Paul M. Emerson, Julius Schachter, Jeremy D. Keenan

**Affiliations:** 1 The Carter Center, Addis Ababa, Ethiopia; 2 Department of Laboratory Medicine, University of California San Francisco, San Francisco, California, United States of America; 3 F.I. Proctor Foundation, University of California San Francisco, San Francisco, California, United States of America; 4 Department of Ophthalmology, University of California San Francisco, San Francisco, California, United States of America; 5 Department of Epidemiology and Biostatistics, University of California San Francisco, San Francisco, California, United States of America; 6 The Carter Center, Atlanta, Georgia, United States of America; University of Washington, United States of America

## Abstract

**Background:**

An important component of the World Health Organization's comprehensive trachoma elimination strategy is the provision of repeated annual mass azithromycin distributions, which are directed at reducing the burden of ocular chlamydia. Knowledge of characteristics associated with infection after mass antibiotic treatments could allow trachoma programs to focus resources to those most likely to be infected with ocular chlamydia.

**Methodology/Principal Findings:**

We monitored 12 communities in rural Ethiopia that had received 3 annual mass azithromycin treatments as part of a cluster-randomized trial for trachoma. One year after the third treatment, a random sample of children from each village received conjunctival examination for follicular trachomatous inflammation (TF) and intense trachomatous inflammation (TI), conjunctival swabbing for chlamydial RNA and DNA, and a household survey. The primary outcome for this study was RNA evidence of ocular chlamydia, which we detected in 41 of 573 swabbed children (7.2%, 95%CI 2.7–17.8). In multivariate mixed effects logistic regression models, ocular chlamydial RNA was significantly associated with ocular discharge (OR 2.82, 95%CI 1.07–7.42), missing the most recent mass azithromycin treatment (OR 2.49, 95%CI 1.02–6.05), having a sibling with ocular chlamydia (OR 4.44, 95%CI 1.60–12.29), and above-median community population (OR 7.81, 95%CI 1.56–39.09). Ocular chlamydial infection was also independently associated with TF (OR 3.42, 95%CI 1.56–7.49) and TI (OR 5.39, 95%CI 2.43–11.98).

**Conclusions/Significance:**

In areas with highly prevalent trachoma treated with multiple rounds of mass azithromycin, trachoma programs could consider continuing mass azithromycin treatments in households that have missed prior mass antibiotic treatments, in households with clinically active trachoma, and in larger communities.

## Author Summary

Trachoma, which is the leading infectious cause of blindness worldwide, is caused by repeated ocular infection with *Chlamydia trachomatis*. Treatment for trachoma includes mass azithromycin treatments to the entire community. The World Health Organization recommends at least 3 rounds of annual mass antibiotic distributions in areas with trachoma, with further mass treatments based on the prevalence of trachoma. However, there are other options for communities that have received several rounds of treatment. For example, programs could continue antibiotic treatments only in those households most likely to have infected individuals. In this study, we performed trachoma monitoring on children from 12 Ethiopian communities one year after a third mass azithromycin treatment, and conducted a household survey at the same time. We found that children were more likely to be infected with ocular chlamydia if they had ocular inflammatory signs or ocular discharge, or if they had missed the preceding antibiotic treatment, had an infected sibling, or came from a larger community. These risk factors suggest that after mass azithromycin treatments, trachoma programs could consider continuing antibiotic distributions to households that have missed prior antibiotic distributions, in households with children who have the clinical signs of trachoma, and in larger communities.

## Introduction

As part of the SAFE strategy (Surgery for trichiasis, Antibiotics, Facial hygiene promotion, and Environmental improvements), the World Health Organization recommends repeated annual mass antibiotic distributions for trachoma, usually with oral azithromycin, followed by reassessment after at least 3 years of SAFE [1]. In areas with highly prevalent trachoma, three treatments are unlikely to be sufficient to eliminate the causative agent, *Chlamydia trachomatis* [2], [3]. In these areas with highly prevalent disease, re-infection rapidly occurs, even after ocular chlamydia has been brought to very low levels with repeated mass azithromycin treatments [4]. The source of re-infection is not entirely clear. It is possible that untreated neighboring communities provide the source of infection, and that travel to or visitors from these untreated communities helps spread infection [5]. Alternatively, it is possible that a reservoir of infection remains in a treated community after mass azithromycin treatments, either because of incomplete antibiotic coverage, or inefficacy of the antibiotic in certain individuals.

It would be helpful for trachoma programs to identify factors associated with being infected with ocular chlamydia after repeated mass azithromycin treatments. Trachoma programs could direct more resources to households with such factors, or could try to improve the status of these factors, in an effort to reduce any reservoirs of chlamydial infection after mass treatments. In this study, we performed trachoma monitoring and household surveys for 12 communities in Ethiopia that had been treated with 3 annual mass azithromycin treatments, to assess which factors are associated with ocular chlamydia after repeated mass antibiotic treatments.

## Methods

### Ethics Statement

This study was approved by the Committee for Human Research at the University of California, San Francisco; the Institutional Review Board at Emory University; and the Ethiopian Science and Technology Commission. The guardians of all study participants gave verbal consent in Amharic; we obtained verbal consent due to the high level of illiteracy in this region. Verbal consent was approved by the institutional review boards, and documented on the field data sheets.

### Study Design

We performed a cross-sectional study of 12 communities in Goncha Siso Enese *woreda*, Amhara Region, Ethiopia to determine risk factors for ocular chlamydia infection after mass azithromycin treatments. The 12 communities had been treated with 3 annual mass azithromycin treatments as part of a cluster-randomized clinical trial for trachoma (clinicaltrials.gov #NCT00322972) [6], [7]. During the trial, we performed an annual population census, followed by a mass azithromycin distribution to all persons aged 1 year and older (single dose of oral azithromycin; 1 g for adults, 20 mg/kg for children). Antibiotic distributors documented whether each individual on the census had received a dose of azithromycin.

### Trachoma Monitoring

In May 2009 (1 year after the third mass azithromycin treatment), we performed monitoring for ocular chlamydia and clinically active trachoma. We chose a random sample of 50 children aged 0–9 years from each of the 12 communities, using a population census that had been performed for the trial 6 months earlier. We examined the upper right tarsal conjunctiva of each child, grading for follicular trachomatous inflammation (TF) and intense trachomatous inflammation (TI) according to the World Health Organization simplified grading scale [8]. Graders were trained at the beginning of the study visit, and only allowed to grade if they achieved sufficient agreement (kappa≥0.6) with a consensus grade from 3 experienced trachoma graders (BA, BDG, TML) regarding the presence of clinically active trachoma (TF and/or TI) on a set of 50 conjunctival photographs. Kappas for clinically active trachoma for the 8 graders in this study ranged from 0.66 to 0.88. We collected 2 swabs of the upper right tarsal conjunctiva: first, a Dacron swab, and then, a swab from the APTIMA-CT Unisex Swab Specimen Collection Kit (Gen-Probe, Inc., San Diego, CA), which was stored in transport media from the same kit. Swabs were transported to the University of California, San Francisco, where the Dacron swabs were processed for chlamydial DNA using AMPLICOR (Roche Diagnostics, Indianapolis, IN), and the APTIMA swabs were processed for chlamydial RNA using APTIMA-CT. In each case, swabs were analyzed as pools of 5 swabs, with individual testing of any positive pools.

### Household Survey

Using the census records, we identified the households of examined children. Trained local health workers and nurses conducted a survey of each identified household in the local language, Amharic. Surveys were performed between 3 and 21 days following the trachoma monitoring. The intended survey respondent was the head of the household or spouse. If either of these persons were not at home, the survey team returned to the household at a different time. If after three visits the head of household or spouse could still not be located, a neighbor was requested to respond to the survey. The survey questions were developed in conjunction with local health workers and nurses, and consisted of questions regarding socioeconomic status, use of antibiotics, availability of latrines and water, and travel. In addition, all children in the household who were present at the time of the survey were examined for ocular discharge (discharge on the eyelashes or eyelids), nasal discharge (discharge on nares, cheeks, or lips), and flies on the face (presence of 1 or more flies on the face during the 3-second period of time after all flies had been shooed away).

### Statistical Methods

The primary outcome in this study was RNA evidence of chlamydial infection, chosen because this is the most sensitive test for chlamydia currently available [9], [10]. We performed univariate mixed effects logistic regression with the presence of chlamydial RNA as the outcome, and community as a random effect. Any risk factors significantly associated with chlamydial infection at *p*<0.05 were included in a multivariate mixed effects logistic regression model, and a backwards stepwise selection process was used until all risk factors in the model were significant at *p*<0.05. We did not include TF or TI as predictors in multivariate models since in communities with hyperendemic trachoma, these clinical signs are most likely a result of chlamydial infection, as opposed to a risk factor for infection. As a sensitivity analysis, we performed similar analyses but included household nested in community as a random effect. As secondary outcomes, we also assessed risk factors for the presence of ocular chlamydia DNA, and clinically active trachoma, defined as TF and/or TI. In the case that the risk factor perfectly predicted the outcome, penalized maximum likelihood regression using Firth's method was used [11]. For any particular analysis, observations with missing data for the outcome or risk factor(s) were omitted. The sample size was based on the underlying clinical trial, which assessed 50 children per community. Analyses were performed with Stata 10 (Statacorp, College Station, TX).

## Results

The 12 communities had a median population of 285 (IQR 212–354). As reported previously, the median prevalence of DNA evidence of ocular chlamydial infection before treatment was 45% (IQR 33–54) and the median prevalence of clinically active trachoma (TF/TI) before treatment was 68% (IQR 55–85) [6]. All 12 communities had received 3 annual mass azithromycin treatments, with antibiotic coverage in children under 10 years of age averaging 80.9% (±13.3%) at the first treatment, 92.1% (±5.0%) at the second treatment, and 87.3% (±11.8%) at the third treatment [6]. We performed trachoma monitoring 1 year after the third mass azithromycin treatment. We examined 583 children under 10 years of age, representing 370 households from 12 communities [7]. We were able to complete a household survey for 575 monitored children, who lived in 364 different households. We could not locate an adult respondent for the remaining 8 children, who lived in 7 different households from 3 of the communities.

As shown in Table 1, interviews were conducted primarily with heads of household (43.0%) or spouse (39.8%), although we did accept responses from neighbors or other community members if heads of household were not at home (17.2%). Heads of household were overwhelmingly male (92.4%), farmers (99.7%), Christian (100%), and without formal education (85.9%). Households were generally characterized by poor access to latrines (28.8% of households had a usable latrine) but good access to water (80.7% of households were within 30 minutes from water). Most households attended market and religious services at least weekly (64.2% and 92.7%, respectively).

**Table 1:** Characteristics of 364 households surveyed one year after a third mass azithromycin treatment.

Household Characteristic	Number*	Proportion (95% CI)
**Survey respondent**		
Head	150/349	43.0% (33.1–53.5%)
Spouse	139/349	39.8% (31.7–48.6%)
Neighbor	54/349	15.5% (9.8–23.6%)
Other	6/349	1.7% (0.4–8.0%)
**Head of household**		
Male Gender	314/340	92.4% (88.0–95.2%)
Christian Religion	357/357	100% (99.0–100%)
Occupation		
Farmer	358/359	99.7% (97.6–99.9%)
Health Extension Worker	1/359	0.3% (0.03–2.4%)
Education		
0 years	104/355	29.30% (17.8–44.3%)
Non-formal education†	201/355	56.6% (41.7–70.5%)
2–10 years	50/355	14.1% (10.4–18.8%)
**Development**		
Distance to water		
<30 minutes	284/352	80.7% (59.1–92.4%)
30–60 minutes	42/352	11.9% (5.7–23.5%)
>1 hour	26/352	7.4% (1.4–31.5%)
Latrine at household		
Yes, usable	104/361	28.8% (18.3–42.3%)
Yes, but unusable	23/361	6.4% (3.3–12.1%)
No	234/361	64.8% (52.2–75.7%)
Electricity in household	0/352	0% (0–1.0%)
**Antibiotic use**		
Any antibiotic use in past 3 months	99/364	27.2% (20.1–35.7%)
**Travel by any member of household**		
Currently	176/364	48.4% (38.0–58.8%)
In past month	360/364	98.9% (97.2–99.6%)
>14 days in past month	170/364	46.7% (31.2–62.9%)
Market visits per month		
1–3	129/360	35.8% (24.1–49.5%)
4	205/360	56.9% (42.2–70.6%)
5–10	26/360	7.2% (4.2–12.2%)
Church visits per month		
1–3	26/356	7.3% (4.1–12.7%)
4	247/356	69.4% (54.8–80.9%)
5–8	55/356	15.5% (7.4–29.5%)
9–30	28/356	7.9% (4.3–14.1%)
**Household visitors**		
Currently	15/364	4.1% (2.3–7.3%)
In past month	110/364	30.2% (19.8–43.3%)

*Denominators less than 364 indicate missing data.

**†:** Refers to a government-sponsored illiteracy campaign.

Of children who underwent trachoma monitoring and a household survey, chlamydial RNA was detected in 41/573 children (7.2%, 95%CI 2.7–17.8), chlamydial DNA in 25/575 children (4.4%, 95%CI 1.7–10.6%), and clinically active trachoma in 247/571 children (43.3%, 34.8–52.1%). TF and TI were independently associated with ocular chlamydia (Table 2). We calculated the predictive values of clinically active trachoma for chlamydial infection at the household level. Of the 200 households in which at least 1 examined child was noted to have TF and/or TI, 28 had at least 1 child with evidence of chlamydial RNA (i.e., household-level positive predictive value 14.0%, 95% CI 6.0–29.3%). In comparison, there were 164 households in which no examined children had either TF or TI, and in 157 of these, no examined child tested positive for chlamydial RNA (i.e., household-level negative predictive value 95.7%, 95%CI 84.2–99.0%).

**Table 2:** Association between clinical signs of trachoma and ocular chlamydial infection after mass azithromycin treatments.

		OR (95%Confidence Interval)*	
Clinical Sign	Proportion (No.)	RNA	DNA
**Model 1** †			
TF only	26.8% (153/571)	1.93 (0.73–5.14)	8.13 (1.61–40.9)
TI only	8.9% (51/571)	2.13 (0.58–7.82)	8.67 (1.31–57.3)
TF+TI	7.5% (43/571)	20.4 (6.93–60.3)	88.9 (15.7–502)
Normal	56.7% (324/571)	Reference	Reference
**Model 2** ‡			
TF	34.3% (196/571)	3.42 (1.56–7.49)	9.18 (2.90–29.03)
TI	16.5% (94/571)	5.39 (2.43–11.98)	10.22 (3.59–29.11)

TF = follicular trachomatous inflammation; TI = intense trachomatous inflammation; RNA = chlamydial rRNA; DNA = chlamydial DNA.

*Multivariate mixed effects logistic regression with either chlamydial RNA or DNA as the outcome, and community as a random effect.

**†:** Clinical signs of trachoma treated as a single categorical variable, with the absence of TF or TI (i.e., normal exam) as the reference; Wald p-value for categorical variable <0.0001 for each outcome.

**‡:** Clinical signs of trachoma treated as separate dichotomous variables.

In total, the 364 households were comprised of 2,079 persons, including 863 children under 10 years of age. Non-programmatic antibiotic use during the preceding 3 months was reported for 123 persons of all ages (5.9%, 95%CI 3.9–8.9%), and 36 children under 10 years of age (4.2% of children, 95%CI 2.9–6.0%). Most persons took a single course of antibiotics, though 11/123 (8.9%, 95%CI 4.0–19.0%) had two courses. Of the 134 antibiotic courses for which a treatment indication was reported, 28 were taken for a respiratory infection, 23 for fever, 18 for diarrhea, 8 for intestinal worms, and 57 for other indications.

The travel patterns of surveyed households are shown in Tables 3 and 4. In general, adults traveled more commonly than children. For household members who traveled, the total amount of time spent away per month was bimodal, with 48.5% of the 1,129 travelers away for 4–8 days, and 25.7% away for 18–22 days. The vast majority of visitors stayed for short amounts of time; 93.9% of the 262 visitors stayed at the household for 3 days or less.

**Table 3:** Travel by household members and visits from outside the community.

	Households		Children < 10 years		Persons ≥ 10 years	
	% (95%CI)	No./Total	% (95%CI)	No./Total	% (95%CI)	No./Total
Household Travelers						
On survey day	48.4% (38.0–58.8)	176/364	4.8% (1.8–12.0%)	41/863	24.5% (19.2–30.8)	298/1216
In past month	98.9% (97.2–99.6)	360/364	14.3% (8.0–24.2%)	123/863	82.7% (76.7–87.5)	1006/1216
Household Visitors						
On survey day	4.1% (2.3–7.3%)	15/364	N/A	0*	N/A	19*
In past month	30.2% (19.8–43.3%)	110/364	N/A	0*	N/A	262*

*Number of visitors to all 364 households; denominator unknown.

N/A = not applicable.

**Table 4:** Travel destination and time spent at destination.

	Children aged < 10 years			Persons aged ≥ 10 years		
Destination	% of Travelers (No.)		Days per Month, mean	% of Travelers (No.)		Days per Month, mean
Market	8.9%	(11/123)	2.64±1.29	70.0%	(704/1006)	2.96±1.28
Church	7.3%	(9/123)	5.44±5.73	66.3%	(667/1006)	4.61±3.63
School	69.1%	(85/123)	18.6±3.68	25.3%	(254/1006)	19.2±3.79
Town	5.7%	(7/123)	2.43±2.15	4.4%	(44/1006)	4.05±7.18
Other	20.1%	(27/123)	2.67±4.61	13.3%	(134/1006)	4.11±7.63

Numbers do not sum to 100% since some household members traveled to multiple destinations.

Table 5 shows the results of univariate analyses for the primary outcome (chlamydial RNA) as well as the secondary outcomes (chlamydial DNA and clinically active trachoma) for the 575 children who received both trachoma monitoring and a household survey. Because having an infected sibling depended on whether a sibling had been selected for trachoma monitoring, we also performed an analysis restricted only to those 375 children who had a sibling monitored at the study visit; chlamydial RNA was still associated with having an infected sibling in this analysis (OR 7.44, 95%CI 2.25–24.56).

**Table 5:** Factors associated with chlamydial infection or active trachoma after mass azithromycin distributions—univariate analyses.

		Odds Ratio (95% Confidence Interval)*		
Factor	Proportion (No.)or Mean ±SD	RNA	DNA	TF/TI
**Individual demographics**				
Age, years	5.2±2.7	0.95 (0.84–1.07)	0.89 (0.77–1.04)	**0.84 (0.79–0.90)**
Male gender	51.9% (298/574)	0.91 (0.45–1.83)	1.03 (0.44–2.38)	**1.50 (1.06–2.12)**
**Individual examination**				
Ocular discharge	15.8% (91/575)	**3.15 (1.27–7.80)**	**3.79 (1.40–10.26)**	**3.51 (2.10–5.86)**
Nasal discharge	53.6% (308/575)	1.38 (0.64–2.98)	0.90 (0.36–2.28)	**1.46 (1.00–2.12)**
Flies on face	15.0% (86/575)	0.92 (0.33–2.53)	1.18 (0.37–3.80)	1.59 (0.97–2.61)
**Individual antibiotic use**				
No mass azithromycin 1 year prior	12.1% (65/536)	**3.07 (1.30–7.26)**	**4.76 (1.83–12.36)**	**1.88 (1.08–3.28)**
No antibiotics in past 3 months	95.5% (549/575)	1.46 (0.17–12.70)	2.58 (0.15–43.49)†	0.62 (0.28–1.40)
**Individual travel**				
Currently	4.5% (26/575)	1.41 (0.27–7.45)	2.40 (0.45–12.75)	0.73 (0.31–1.75)
>7 days in past month	9.7% (56/575)	1.59 (0.51–4.89)	1.42 (0.37–5.52)	0.83 (0.45–1.50)
>14 days in past month	9.0% (52/575)	1.79 (0.56–5.74)	1.62 (0.40–6.50)	0.72 (0.38–1.36)
**Household Sociodemographics**				
Number in household	5.9±1.8	0.99 (0.80–1.23)	0.95 (0.73–1.23)	0.91 (0.83–1.01)
Presence of sibling <5 years	56.0% (322/575)	2.03 (0.97–4.27)	1.79 (0.73–4.39)	0.90 (0.64–1.28)
Number of siblings <5 years	0.7±0.7	**1.67 (1.02–2.72)**	1.61 (0.90–2.88)	0.96 (0.75–1.23)
Distance to water ≥30 minutes	21.1% (117/575)	0.48 (0.09–2.65)	0.33 (0.03–3.17)	0.61 (0.36–1.02)
No usable latrine	72.0% (412/572)	1.35 (0.57–3.20)	3.37 (0.93–12.15)	1.20 (0.80–1.80)
No education, head of household	85.8% (483/563)	1.96 (0.64–6.01)	2.48 (0.55–11.14)	1.40 (0.84–2.34)
Survey completed by neighbor/other	16.2% (93/575)	0.96 (0.41–2.24)	1.75 (0.69–4.44)	1.26 (0.79–2.02)
**Sibling factors**				
Sibling with chlamydial RNA	4.2% (24/575)	**5.05 (1.86–13.73)**	**13.82 (4.32–44.18)**	1.92 (0.78–4.71)
Sibling with TF/TI	33.0% (190/575)	1.30 (0.64–2.64)	1.95 (0.84–4.53)	1.32 (0.92–1.90)
No mass azithromycin to sibling 1 y prior	22.4% (129/575)	**2.21 (1.04–4.69)**	2.43 (0.99–5.97)	0.76 (0.49–1.19)
**Antibiotic use by anyone in household**				
No antibiotics in past 3 months	71.8% (413/575)	1.09 (0.48–2.46)	1.46 (0.51–4.16)	0.92 (0.62–1.36)
**Travel by anyone in household**				
Currently	48.0% (276/575)	1.72 (0.83–3.57)	**3.44 (1.30–9.14)**	1.14 (0.79–1.63)
>7 days in past month	81.9% (471/575)	1.76 (0.57–5.47)	11.94 (0.72–197.7)†	0.94 (0.59–1.50)
>14 days in past month	47.3% (272/575)	1.01 (0.47–2.15)	0.88 (0.35–2.19)	0.81 (0.55–1.19)
Market >4 times per month	7.4% (42/571)	1.25 (0.38–4.10)	1.62 (0.43–6.16)	1.52 (0.79–2.94)
Church >4 times per month	24.3% (137/564)	1.07 (0.44–2.57)	0.38 (0.10–1.43)	0.82 (0.53–1.26)
**Household visitors**				
Currently	4.4% (25/575)	1.79 (0.33–9.63)	3.15 (0.58–17.10)	1.12 (0.48–2.64)
In past month	31.7% (182/575)	1.86 (0.88–3.93)	**3.35 (1.31–8.52)**	**1.50 (1.01–2.21)**
Number of visitors in past month	0.7±1.3	1.22 (0.95–1.57)	**1.45 (1.09–1.94)**	1.07 (0.93–1.24)
**Village factors**				
Population (100s of persons)	3.13±1.15	2.00 (0.89–4.52)	1.94 (0.83–4.53)	1.07 (0.82–1.40)
Population >285 persons	51.5% (296/575)	**9.42 (1.64–53.94)**	**12.03 (1.99–72.81)**	1.47 (0.82–2.66)
Pre-treatment prevalence CT >45%	50.8% (292/575)	0.78 (0.09–6.77)	0.87 (0.11–6.80)	1.42 (0.78–2.58)
Pre-treatment prevalence TF/TI >68%	47.3% (272/575)	2.62 (0.36–19.04)	1.69 (0.23–12.20)	1.05 (0.56–1.97)
1^st^ round antibiotic coverage <90%	83.7% (481/575)	3.18 (0.16–63.90)	4.05 (0.19–87.55)	0.97 (0.42–2.26)
2^nd^ round antibiotic coverage <90%	30.8% (177/575)	0.83 (0.09–7.96)	0.57 (0.06–5.18)	1.05 (0.53–2.05)
3^rd^ round antibiotic coverage <90%	48.0% (276/575)	**8.08 (1.46–44.87)**	**8.02 (1.60–40.25)**	1.68 (0.96–2.93)

CT = *Chlamydia trachomatis*, as detected by AMPLICOR; TF = follicular trachoma; TI = intense inflammatory trachoma; RNA = chlamydial rRNA; DNA = chlamydial DNA.

*Univariate mixed effects logistic regression with either chlamydial RNA, chlamydial DNA, or clinically active trachoma (TF/TI) as the outcome, and community as a random effect. Variables summarized as means were analyzed as continuous variables. Odds ratios with *p*<0.05 shown in bold.

**†:** Penalized maximum likelihood regression using Firth's method; used because the risk factor was present in all DNA+ cases.

In multivariate models (Table 6), chlamydial RNA retained an association with ocular discharge (OR 2.82, 95%CI 1.07–7.42), missing the most recent mass azithromycin treatment (OR 2.49, 95%CI 1.02–6.05), having a sibling with ocular chlamydia (OR 4.44, 95%CI 1.60–12.29), and community population above the median of 285 (OR 7.81, 95%CI 1.56–39.09). Analysis of the secondary outcomes largely supported the results of the primary chlamydial RNA outcome (Tables 5 and 6). As a sensitivity analysis, we performed mixed effects logistic regression models with household nested in community as a random effect. The results of these analyses were similar to those shown in Table 5 for chlamydial RNA and TF/TI, but the chlamydial DNA data did not support this model (data not shown).

**Table 6:** Factors associated with chlamydial infection or active trachoma after mass azithromycin distributions—multivariate analyses.

	Odds Ratio (95% Confidence Interval)*		
Risk factor	RNA	DNA	TF/TI
**Individual Factors**			
Age, per year			0.85 (0.80–0.91)
Male gender			1.46 (1.02–2.11)
Ocular discharge	2.82 (1.07–7.42)	4.69 (1.37–16.04)	3.23 (1.90–5.49)
No mass azithromycin 1 year prior	2.49 (1.02–6.05)	3.78 (1.27–11.24)	
**Household Factors**			
Sibling with chlamydial RNA	4.44 (1.60–12.29)	9.86 (3.06–31.73)	
Any household members currently absent		3.42 (1.19–9.89)	
Any visitors in the past month			1.71 (1.13–2.59)
Number of visitors in past month		1.49 (1.05–2.11)	
**Village Factors**			
Population >285 persons	7.81 (1.56–39.09)	9.74 (1.86–51.06)	

RNA = chlamydial rRNA; DNA = chlamydial DNA; TF = follicular trachomatous inflammation; TI = intense trachomatous inflammation.

*Multivariate mixed effects logistic regression with either chlamydial RNA, chlamydial DNA, or clinically active trachoma (TF/TI) as the outcome, and community as a random effect.

## Discussion

We showed that after 3 repeated mass azithromycin treatments, the factors most strongly predictive of ocular chlamydial RNA were ocular discharge, missing the previous mass azithromycin treatment, having a sibling infected with ocular chlamydia, and living in a larger community. Even after 3 mass treatments, the clinical signs of trachoma were strongly associated with chlamydial infection. These findings were confirmed with analyses using ocular chlamydial DNA as the outcome, and were robust in models that accounted for community and household clustering.

Few studies have assessed for risk factors of ocular chlamydial infection before or after mass azithromycin treatments. Studies conducted before mass treatments have not shown consistent associations, though various studies have suggested the importance of unclean faces, age, household fly density, infected siblings, and absence of a latrine [5], [12], [13]. The few studies conducted after mass azithromycin treatments have generally found that missing a previous mass azithromycin treatment and younger age are associated with chlamydial infection, though individual studies have also implicated the absence of a latrine, travel, and the number of infected and untreated children per household [5], [14], [15]. Previous studies have assessed for DNA evidence of ocular chlamydial infection. In contrast, our primary outcome was ocular chlamydial RNA—the most sensitive test for chlamydia currently available [9], [10].

The multivariate analysis revealed several risk factors for chlamydial RNA after repeated mass treatments. The association between missing the previous mass azithromycin treatment and ocular chlamydial infection confirms the importance of the Antibiotic component of the SAFE strategy. The association between ocular discharge and chlamydial infection seems to indicate that unclean faces are an important risk factor for infection even after mass treatments [16]. However, it is also possible that ocular discharge is not on the causative pathway for ocular chlamydial infection, but is simply a result of being infected. The association between above-median community population and ocular chlamydia could indicate that chlamydial transmission is more likely in crowded communities, or could simply reflect the difficulty for chlamydial infection to fade away in larger communities [17]. This result should be interpreted with caution, since the population variable was based on only 12 villages. It is important to note that although we used multivariate analyses, the observational study design cannot rule out the possibility that the observed associations are the result of unmeasured confounding.

Consistent with previous reports, we were unable to show that accessibility to water or latrine status were associated with either ocular chlamydia or clinical trachoma after mass antibiotics [5], [12], [13]. Although a previous study did find that lack of a latrine was associated with ocular chlamydial infection after mass azithromycin treatment, that study found that the relationship became weaker over time, and by 12 months after the mass treatment (analogous to this study), was no longer significant [5]. It is possible that our inability to detect an association with the E components of the SAFE strategy in the current study is a function of an insufficient sample size, or misclassification during the survey, or an insufficient follow-up period. Nonetheless, across several studies, missing mass antibiotic treatments does seem to be the most important predictor of chlamydial infection and trachoma, at least in the short term. Further investigation into the role of missed azithromycin treatments, including ways to improve coverage of mass treatments, is warranted.

Our results provide evidence that after repeated mass azithromycin treatments, trachoma programs may be able to target antibiotic treatments to those most likely to be infected. For example, TF and TI were independently associated with ocular chlamydial RNA after 3 mass treatments—not surprising, since infection causes the clinical signs of trachoma. Moreover, having an infected sibling was significantly associated with ocular chlamydial infection. This is consistent with previous reports that have shown that ocular chlamydial infection clusters by household [18], [19], [20]. Taken together, these results suggest that treating the households of children with clinically active trachoma, as the WHO has suggested in the past, may be a reasonable way to target those individuals most likely to be infected after repeated rounds of mass azithromycin have already been distributed [21]. In this study, treatment targeted to households in which any child was observed to have clinically active trachoma would have resulted in antibiotic distributions to only 55% (200/364) of households, but still would have covered 80% (28/35) of households with ocular chlamydia. However, the feasibility and cost-effectiveness of such a strategy remains to be determined, since targeting households with clinically active trachoma would require examinations of all children in the community. A simpler strategy might be to continue mass azithromycin treatments in areas with highly prevalent trachoma. In fact, the WHO now recommends 5 rounds of mass treatment for areas with hyperendemic trachoma, a strategy that would likely benefit the communities in this study [22].

As a secondary outcome, we assessed risk factors for having a positive chlamydial DNA test. These results confirmed the risk factors found for the primary RNA outcome, and also suggested that travel outside the community, or hosting a visitor from outside the community, may be associated with ocular chlamydial infection. Travel to and from outside communities could re-introduce ocular chlamydia into a treated household. Other studies have similarly found that travel could be an important risk factor for trachoma [5], [23]. Trachoma programs with knowledge of major migration episodes may choose to wait until after the travel has occurred before scheduling a mass distribution of azithromycin, or treat a large enough area in order to mitigate this potential problem. In addition, given that most travel was done over short periods of time, trachoma programs may increase coverage to most travelers simply by returning to households with absent members in several days time.

We also assessed for risk factors of the clinical signs of trachoma, TF and TI, as a secondary outcome. Most previous studies that have assessed for risk factors for clinically active trachoma have been conducted before mass azithromycin treatments have been initiated. In these studies, several risk factors have consistently been associated with clinically active trachoma, including indicators of poor face hygiene such as nasal discharge and the presence of flies [13], [24], [25], [26], [27], [28], [29], household fly density [12], [25], [28], [30], [31], distance to water [27], [32], [33], [34], [35], [36], absence of a latrine [32], [34], [37], and number of children per household [27], [37], [38], [39]. Fewer studies have reported risk factors for clinically active trachoma after mass antibiotic treatments. These studies have generally demonstrated that younger age, lack of latrines, unclean faces, and missed mass azithromycin treatments are associated with clinically active trachoma [14], [40], [41]. In this study, conducted after 3 annual mass azithromycin distributions, clinically active trachoma was associated with younger age and ocular discharge, two commonly reported risk factors. In addition, active trachoma was associated with having a visitor in the past month, and male gender. The significance of the association between TF/TI and male gender is unclear, as ocular chlamydial infection was not more common among boys. Past studies have more frequently demonstrated an association between clinically active trachoma and female gender [12], [26], [34], [42], though several studies have noted more clinically active trachoma among boys [38], [40]. The generalizability of this finding is uncertain, and may simply reflect the specific sample of individuals in this study.

In both univariate and multivariate analyses, the results for the primary RNA outcome were almost always confirmed by the results for the secondary DNA outcome, but often differed from the results of the TF/TI outcome. This likely occurred because the RNA-based and DNA-based tests are related tests, in that both are testing for genetic evidence of chlamydia. The clinical examination, on the other hand, is a test for conjunctival inflammation, and is often discrepant with laboratory tests [43], [44], [45]. In fact, roughly 43% of children had TF/TI in this study, whereas only 7% had RNA evidence of chlamydial infection, consistent with other reports that have shown that the clinical signs of trachoma persist for many months after chlamydial infection has been cleared [46], [47]. Nonetheless, there were 2 factors that were associated with all 3 outcomes: ocular discharge and missing the previous mass azithromycin treatment. The role of ocular discharge as a risk factor is unclear, since it may simply be a result of infection. However, that missing a previous mass azithromycin treatment was associated with all 3 outcomes suggests that this is an important trachoma risk factor, and suggests trachoma programs should make efforts to enhance antibiotic coverage.

This study has several limitations. We chose a random sample of children from each community, and therefore did not assess the clinical or infectious status of all children in surveyed households. This sampling strategy limited our sample size, which did not allow us to assess for the presence of weaker associations. Due to the observational study design, we are unable to comment on antecedent-consequent relationships. Other limitations of surveys apply, such as the potential for recall bias and misclassification errors.

In conclusion, we showed that after 3 annual mass azithromycin treatments in a region of Ethiopia with highly prevalent trachoma, ocular chlamydial RNA was associated with missing the previous mass antibiotic treatment, ocular discharge, larger community size, having an infected sibling, and the clinical signs of trachoma (TF and/or TI). These findings suggest that (1) maximizing antibiotic coverage and promoting face washing are important goals for trachoma programs; (2) larger communities may require more prolonged treatment compared to smaller communities; and (3) after repeated mass azithromycin treatments, trachoma programs could consider antibiotic distribution strategies that target children with clinically active trachoma and their siblings. Further research into the factors associated with chlamydial infection after repeated mass azithromycin distributions will be helpful to guide trachoma program activities after mass azithromycin distributions have begun.

## Supporting Information

Checklist S1 **STROBE checklist.**
(DOC)
